# A Comparative Study between Knocked-Down Aligned Carbon Nanotubes and Buckypaper-Based Strain Sensors

**DOI:** 10.3390/ma12122013

**Published:** 2019-06-23

**Authors:** Ana Santos, Luís Amorim, João Pedro Nunes, Luís Alexandre Rocha, Alexandre Ferreira Silva, Júlio César Viana

**Affiliations:** 1 IPC/i3N-Institute for Polymers and Composites, University of Minho, 4800-058 Guimarães, Portugal; luis.amorim@dep.uminho.pt (L.A.); jpn@dep.uminho.pt (J.P.N.); jcv@dep.uminho.pt (J.C.V.); 2 CMEMS—Center for MicroElectroMechanical Systems, University of Minho, 4800-058 Guimarães, Portugal; lrocha@dei.uminho.pt (L.A.R.); asilva@dei.uminho.pt (A.F.S.)

**Keywords:** aligned CNTs, buckypaper, knocked-down CNT, strain sensor

## Abstract

Carbon nanotubes (CNTs) are one of the most promising materials in sensing applications due to their electrical and mechanical properties. This paper presents a comparative study between CNT Buckypaper (BP) and aligned CNT-based strain sensors. The Buckypapers were produced by vacuum filtration of commercial CNTs dispersed in two different solvents, N,N-Dimethylformamide (DMF) and ethanol, forming freestanding sheets, which were cut in 10 × 10 mm squares and transferred to polyimide (PI) films. The morphology of the BP was characterized by scanning electron microscopy (SEM). The initial electrical resistivity of the samples was measured, and then relative electrical resistance versus strain measurements were obtained. The results were compared with the knocked-down vertically aligned CNT/PI based sensors previously reported. Although both types of sensors were sensitive to strain, the aligned CNT/PI samples had better mechanical performance and the advantage of inferring strain direction due to their electrical resistivity anisotropic behavior.

## 1. Introduction

From aerospace to microelectronic applications, the growing demand for multifunctional materials with a set of outstanding properties put carbon nanotubes (CNTs) on the map of the most promising ones [1,2]. While CNTs mechanical performance and piezoresistive response make them suitable for sensing applications, the electric anisotropy of aligned CNTs can be used to infer strain directions [3]. This nanomaterial can be incorporated into polymer-based sensors in several ways:

(1) As conductive fillers in polymer nanocomposites [4,5], through mechanical mixing and deposition methods, such as coating, dip casting, and filtration, among others;
(2) As Buckypaper films (BP), produced by dispersion and deposition methods, such as vacuum filtration (the most common method), drop casting, or hot-press compression, among others [6,7];
(3) As vertically aligned CNT forests (VA-CNTs), synthesized by chemical vapor deposition (CVD), incorporated into polymeric matrices and substrates [8,9]; and
(4) As vertically aligned CNT forests (VA-CNTs), synthesized by laser-oriented deposition (LOD) method directly into the matrices, allowing the incorporation of CNTs due to the covalent bounds formed during the process [10]. Furthermore, a dramatic change of materials resistivity via LOD application is reported in the literature [11].

Despite being widely adopted, these methods have some manufacturing limitations regarding the homogeneity of CNT dispersion, the impregnation efficiency, and CNT alignment [12,13]. Moreover, solutions for these drawbacks can be time consuming and complex.

CNT Buckypapers, for instance, have their electrical conductivity and mechanical performance intimately dependent upon the homogeneity of the CNT dispersion [14]. To accomplish that, appropriated surfactants (whose removal can be time-consuming) and some extra manufacturing steps have to be considered. Several authors [13,15,16] produced BP-based sensors with high sensitivity and found a linear dependence between the relative electric resistance and the applied strain. Moreover, some authors studied the effect of CNT sonication parameters [17] in the quality of CNT dispersion and the use of different solvents [18,19] on the performance of the BP-based sensors, which highly depends on the homogeneity of the BP. However, the advantages that can rise from the CNT alignment in a sensor are always borne in mind.

Several techniques for CNT alignment [8,20,21,22], namely, by mechanical stretching, electric and magnetic fields, flow assisted, drawn from VA-CNT forests, fall short due to their complexity. Alternatively, VA-CNTs, widely produced by CVD, can be knocked down and form a kind of aligned CNT Buckypaper. Recently, this technique was used to produce microheaters [23], to improve inter-laminar facture toughness on laminated composites [24], and to develop CNT/polymer strain sensors [3]. In this latter case, a patch of knocked-down VA-CNTs was laid down over a polymeric film substrate, and four conductive electrodes of Ag ink were placed for electrical connections, allowing the measurement of electrical resistance over orthogonal axes.

In this work, two types of CNT/polymer strain sensors are compared, based on:

- Buckypapers, in which CNTs are randomly dispersed with two different solvents and show an isotropic electrical behavior, and;
- Knocked-down VA-CNTs, which are highly aligned in one direction and show anisotropic electrical properties (and were developed in our previous work [3]).

The aim is to assess the effect of the isotropic/anisotropic electrical properties on strain sensor behavior. For this purpose, commercial multi-wall carbon nanotubes (MWCNTs) were dispersed in N,N-Dimethylformamide (DMF) and ethanol, which were chosen in order to avoid the use of organic solvents (e.g., Triton X) and, thus, extra washing steps, and then vacuum filtrated to form freestanding Buckypapers, BP_DMF_ and BP_ETOH_, respectively. After being transferred to polyimide (PI) films, relative electrical resistance and Gauge factor versus strain measurements were obtained. The results are compared with the knocked-down VA-CNT/PI-based sensor.

## 2. Materials and Methods

### 2.1. Aligned CNT/PI Samples Preparation

VA-CNTs were synthesized via CVD, at 750 °C, with a flown gas mixture of ethylene/hydrogen/helium (100/200/55 sccm), in a 10 × 10 mm size silicon patch, previously patterned with Fe/Al_2_O_3_ catalyst. The CNT forests were manually knocked down onto PI films by a 10 mm diameter rod. Also, a silver conductive epoxy adhesive (8330S from MG Chemicals) was used as electrodes between the samples and the copper wires and placed at the corners of the CNT patch. A detailed description of this procedure can be found can elsewhere [3].

### 2.2. CNT Buckypaper Samples Preparation

**Figure 1:**
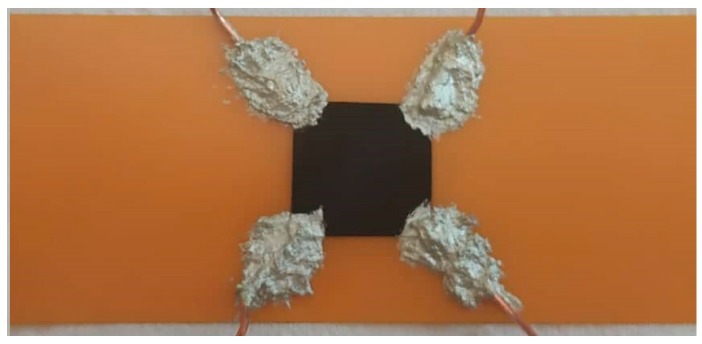
Appearance of a test sample.

Vacuum filtration was the method used to produce the CNT Buckypapers with different dispersing agents. First, 0.025 g of commercial multiwall carbon nanotubes (NC7000TM from Nanocyl) were dispersed in 100 mL of DMF. The solution was stirred for 5 h and left in an ultrasonic bath (CREST ultrasonics, 240 V, 50/60 Hz) for 2 h [6]. Also, 0.025 g of CNTs were dispersed in 50 mL of ethanol with an ultrasonic tip (Hielscher UP200Ht) at 50% speed during 30 min [18]. The dispersions were filtrated through a porous nylon membrane (45 µm), washed with Millipore water, and then dried at 60 °C. The freestanding Buckypapers, BP_DMF_ and BP_ETOH_, respectively, were peeled off from the membrane and 10 × 10 mm squares were cut off and transferred onto the center of a polyimide film, PI (75 µm Kapton MP film). To measure the electrical resistivity of the samples, a silver conductive epoxy adhesive (8330S from MG Chemicals) was used as electrodes, as shown in Figure 1.

### 2.3. SEM Analysis

In order to characterize the surface morphology of the obtained CNT Buckypapers and the alignment of the knocked-down VA-CNTs, a scanning electron microscopy (SEM) analysis was carried out in a NanoSEM-200 apparatus from FEI Nova (FEI Europe, Eindhoven, The Netherlands).

### 2.4. Electrical Resistivity versus Strain Measurements

A MATLAB software (R2018a, Mathworks, Natick, MA, USA) was used to determine the electrical properties of the samples with an adapted Van der Pauw method. The electrical resistivity, *ρ*, of the BP and aligned CNT-based sensors were calculated by Equation (1):

(1) $$\rho =R_{s}d$$

where *d* is the average thickness of the BP or of the aligned CNT patch, and the *R_S_* is a sheet electrical resistance experimental value obtained by a simple adaptation of a Van der Pauw equation (Equation (2)):

(2) $$e^{-\pi R_{vertical}/R_{s}}+e^{-\pi R_{horizontal}/R_{s}}=1$$

where $R_{vertical}$ and $R_{horizontal}$ are the means of the electrical resistance experimental values obtained in the strain and opposite directions, respectively. The electrical resistance values in axial strain and opposite (transverse) directions, *R_strain_* and *R_opp_*, respectively, were determined using $R_{strain}R_{opp}=R_{s}{}^{2}$ and $R_{strain}/R_{opp}\;$values. Specifically, $R_{strain}/R_{opp}$ was obtained from Equation (3):

(3) a2bRstrain/Ropp=∫0π/2dφ1−k2(sinφ)2∫0π/2dφ1−(sinφ)2+k2(sinφ)2

where *a* and *b* are the CNT patches square dimensions, which varies with the deformation of the polymeric film. Initially, *a_0_* = *b_0_* = 10 mm, and upon deformation increment, in strain direction: *a* = *a_0_* + Δ*l* (Δ*l* is the elongation of the film considering the perfect adhesion between the electrodes and the film), and in the transverse direction: *b = b_0_ − vεl_t0_* considering the Poisson ratio, *v*, of 0.34 for PI film, where ε is the mechanical strain and *l_t0_* is the initial width of the film. The *k* value is obtained from Equation (4):

(4) $$\alpha =\frac{V_{DC}/I_{AB}}{V_{BC}/I_{AD}}=\frac{ln\frac{4/k}{{\left(1/k+1\right)}^{2}}}{ln\frac{{\left(1/k-1\right)}^{2}}{{\left(1/k+1\right)}^{2}}}$$

where *I_AB_* and *I_AD_* are the injected currents in the CNT patch in two different directions and *V_DC_* and *V_BC_* are the respective voltages measured. A more detailed description can be found elsewhere [3].

The samples’ process of measuring the electrical resistivity versus strain was quite similar to that described previously [3]. The BP_DMF_/PI and BP_ETOH_/PI samples were strained using a manual microtester and the elongation of the samples (∆*l*) was measured by a digital calliper (Mitutoyo). The relative electrical resistance, ∆*R/R_0_*, and gauge factor, GF (Equation (5)), which is the ratio between the relative electrical resistance and mechanical deformation, *ε* = ∆*l/l_0_*, were evaluated upon increased strain levels:

(5) $$GF=\frac{\Delta R/R_{0}}{\varepsilon }$$

where *R_0_* is the initial (unstrained sample) electrical resistance. It is important to note that the GF values in the opposite direction of the applied strain were calculated using transverse deformation values and plotted versus axial strain deformation.

## 3. Results

### 3.1. SEM Analysis

**Figure 2:**
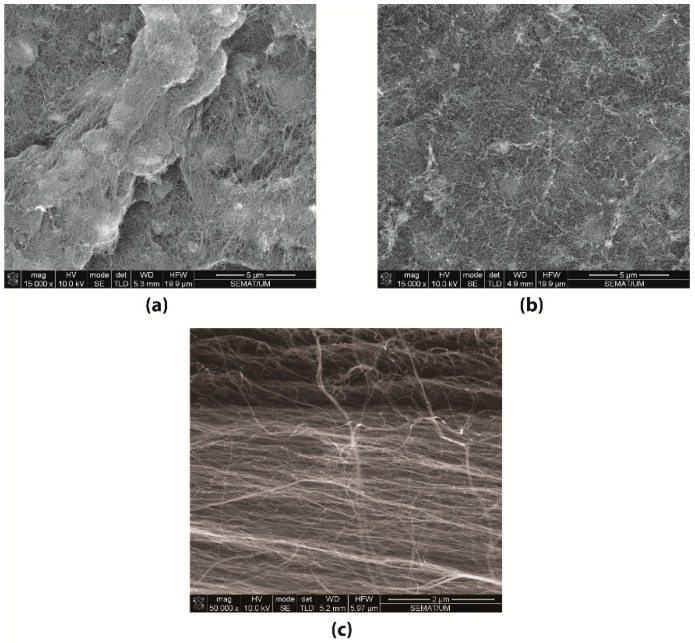
SEM images of the BP_DMF_ (**a**), BP_ETOH_ (**b**), and knocked-down carbon nanotubes (CNTs) (**c**) samples.

From the SEM images of the BP_DMF_ and BP_ETOH_ samples shown in Figure 2a,b, the CNTs appeared to be randomly dispersed within the Buckypapers. However, considerable CNT agglomerates can be seen in the BP_DMF_ samples in comparison to the more homogenous surface of the BP_ETOH_ samples, which was also confirmed visually. The nonhomogeneous CNT dispersion observed in those BP_DMF_ samples was what led to the production of Buckpapers using ethanol, BP_ETOH_.

To assess the alignment of the knocked-down CNTs, an SEM analysis was also performed. As shown in Figure 2c, the CNTs were aligned almost in the horizontal direction (parallel to the PI substrate), despite some squashing of the sample due to the knocked down process.

### 3.2. Electrical Resistivity versus Strain Measurements

**Figure 3:**
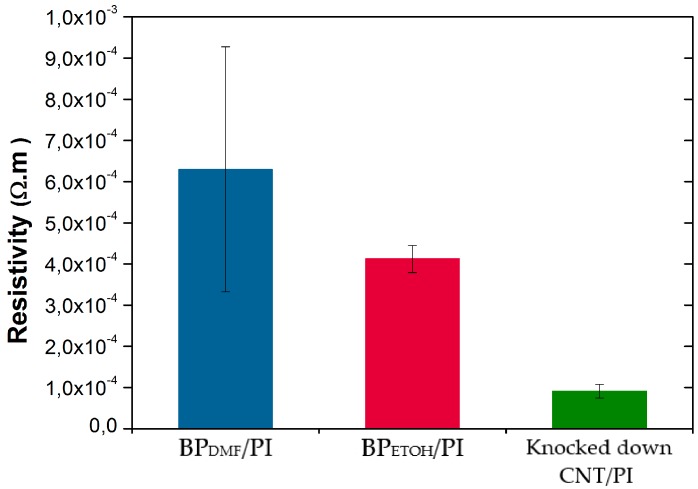
Electrical resistivity values of the BP_DMF_, BP_ETOH_, and knocked-down CNT samples.

As plotted in Figure 3, the initial electrical resistivity of the BP_DMF_/PI was higher than BP_ETOH_/PI samples and showed more variability, probably due to the nonhomogeneous CNT distribution (as revealed by the SEM images), which compromises the number of conductive paths in the CNT network, thus increasing the electrical resistance. As expected, the knocked-down VA-CNT/PI samples showed the lowest electrical resistivity value due to the high CNT alignment. All these values were according to the ones reported elsewhere [6,18,25].

**Figure 4:**
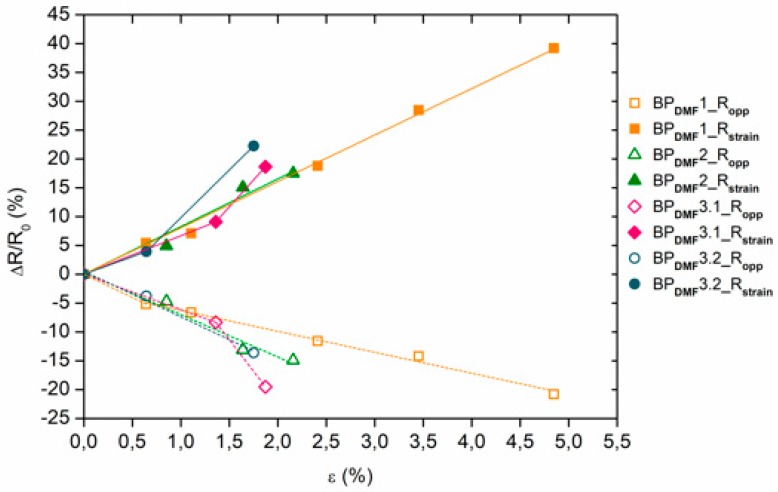
Relative electrical resistance ∆R/R_0_ as function of the strain ε for the BP_DMF_/PI samples (R_strain_ and R_opp_ values in axial strain and opposite (transverse) directions, respectively).

**Figure 5:**
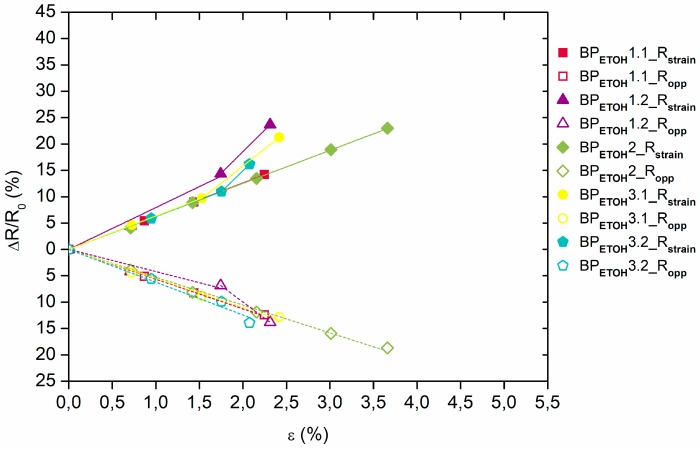
Relative electrical resistance ∆R/R_0_ as function of the strain ε for the BP_ETOH_/PI samples (R_strain_ and R_opp_ values in axial strain and opposite (transverse) directions, respectively).

As shown in Figure 4 and Figure 5, the relative electrical resistance values in the strain direction of the BP_DMF_/PI and BP_ETOH_/PI samples always increased, almost linearly, with the strain increments, while they decreased in the opposite direction. The BP_DMF_/PI samples that reached a mechanical breaking point at approximately 2% of deformation with a sudden steeper slope presented relative electrical resistance values between 17% and 22% in the strain direction and had similar behavior in the opposite direction. The relative electrical resistance of one of the BP_DMF_/PI samples reached, with a steady slope, values of 40% and −20% in strain and opposite directions, respectively, and higher deformations at break of 5%. These differences between relative electrical resistances in opposite directions are probably due to the Poisson contraction effect. In a similar way, most of the BP_ETOH_/PI samples reached a deformation at break below 2.5%, with a sudden steeper slope, presenting relative electrical resistance values between 14% and 24% in strain direction and had also similar behavior in the opposite direction. One sample showed higher deformations at break of almost 4%, reaching a steady slope, approximately, 23% and −19% of relative electrical resistance in strain and opposite directions, respectively.

These results are according to the CNT–CNT junctions model for electrical conduction [26]: When stretched in the strain direction, the number of CNT–CNT junctions decreases with strain, resulting in a linear increase of the electrical resistance, while in the opposite direction, this number increases due to the Poisson contraction effect and the electrical resistance decreases.

**Figure 6:**
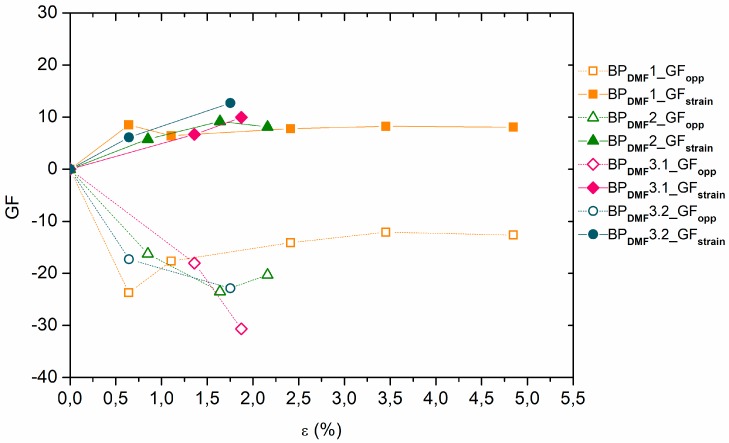
Gauge factor (GF) versus strain ε for the BP_DMF_/PI samples (GF_strain_ and GF_opp_ values in axial strain and opposite (transverse) directions, respectively).

**Figure 7:**
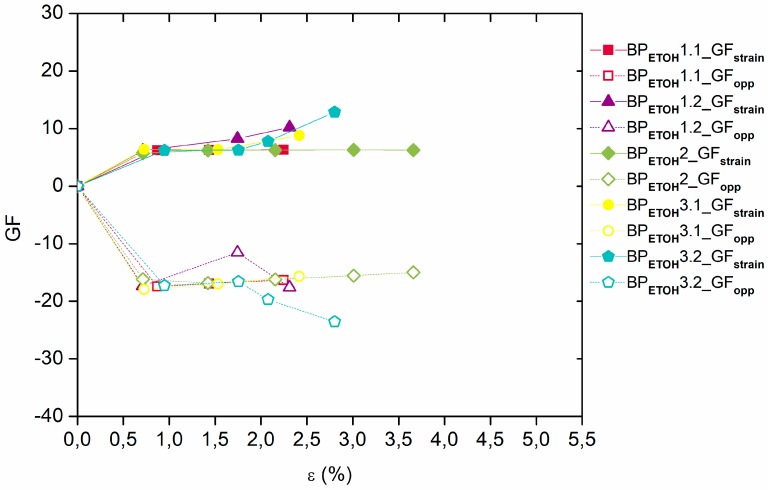
Gauge factor (GF) versus strain ε for the BP_ETOH_/PI samples (GF_strain_ and GF_opp_ values in axial strain and opposite (transverse) directions, respectively).

As shown in Figure 6, the values of the gauge factor, GF, of the BP_DMF_/PI samples are almost constant between 7.7 and 9.4 in the strain direction, while in the opposite direction, a higher variability was observed, with values approximately between −24.3 and −16 (Table 1). Regarding BP_ETOH_/PI samples (Figure 7), the values of the gauge factor, GF, are also almost constant in the axial strain direction, varying between 7.2 and 8.2, while in the opposite direction, a higher variability was observed, with values between −17.9 and −15.5 (Table 1). Both BP sensors showed higher sensitivity in opposite direction of strain (transverse) due to the fact that the GF values in that direction were calculated using transverse deformation despite being plotted against axial deformation. Therefore, a correction of these values with the Poisson ratio, ν, was presented in Table 1 and a similar sensitivity is then observed in the two different directions. Although, in the BP_ETOH_/PI samples, the CNTs were much more homogeneously dispersed than in BP_DMF_/PI, the slight differences observed in the electrical properties are probably due to the presence of random agglomerates in both types of Buckypapers, as already reported elsewhere [19].

**Figure 8:**
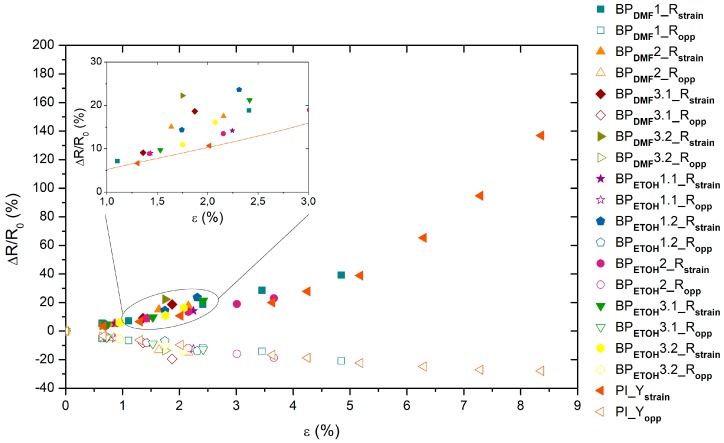
Relative electrical resistance ∆*R/R_0_* as function of the strain ε for the BP_DMF_/PI, BP_ETOH_/PI and knocked down VA-CNT/PI sample strained in direction of CNT alignment (PI_Y_strain_ and PI_Y_opp_ represent the value in strain and opposite directions, respectively).

In Figure 8, the previous results are compared with the values for the knocked-down VA-CNT/PI-based sensor. Both sensor types are strain sensitive even at low strains, presenting relative electrical resistance values higher than some reported in the literature [6] (see also Table 2). The Buckypapers-based sensors show, with a linear trend, higher relative electric resistance values and, consequently, a higher sensitivity between approximately 1.5% and 2.5% of deformation (inset graph in Figure 8). However, the aligned CNT/PI sensors present an almost exponential trend, due to tunneling effect conductive mechanisms that become dominant, and thus, higher sensitivity to higher strains, and as seen in Table 1, their GF values in strain and opposite directions highlighted the sensitive differences due to CNT alignment.

In Table 2, the GF of the CNT-based sensors of this work are compared with others in the literature. The different fabrication methods and polymer matrices are also referred to.

## 4. Conclusions

Carbon nanotube Buckypaper-based strain sensors were successfully produced. Morphological differences between BP_DMF_ and BP_ETOH_ were observed by SEM analysis, specifically the presence of CNT agglomerates in BP_DMF_. However, these CNT non-homogeneities did not seem to significantly influence the electrical properties, which did not present considerable variability between the two types of BP. Nevertheless, BP_DMF_-based sensors showed slightly higher GF values (7.7–9.4) compared with BP_ETOH_-based sensors (7.2–8.2). These values are somewhat lower in the transverse direction, showing the quasi-isotropic electrical behavior of BP-based sensors. Despite showing higher relative electrical resistances (at low strain level) compared to the knocked-down VA-CNT/PI-based strain sensors, these latter showed higher mechanical performance and improved electrical properties. The deformation at break are much higher (8.4%) as compared with BP sensors (2.5%–2.7%). The GF of the knocked-down VA-CNT-based sensors are the highest (16.4) when stretched in the CNT direction. On the opposite direction, GF values are remarkably lower, evidencing the unique electrical anisotropic behavior of VA-CNT/PI-based sensors. Although the electrical conduction mechanisms in BP and knocked-down CNTs are dependent on the number of CNT–CNT junctions, the alignment of CNTs causes a variation of these numbers in opposite directions. Moreover, these aligned CNT sensors also show a tunneling effect that becomes predominant at higher strains. In the case of the isotropic BP sensors, the number of CNT–CNT junctions are identical in orthogonal directions, resulting in similar relative electrical resistance. Specifically, for VA-CNT sensors, in the CNT alignment direction, this number decreases with strain, increasing the electrical resistance, whereas in the opposed direction, this number increases, decreasing the electrical resistance at a slower rate.

This electrical anisotropic behavior of VA-CNT/PI sensors can be a huge advantage, potentially allowing us to identify the direction of applied strain. Their higher deformation capabilities also allow their use as large strain sensors, despite the loss of the linearity behavior.

## Figures and Tables

**Table 1:** Summary of the sensitivity, electrical properties, and deformation at break (ε_b_) of the BP and knocked-down VA-CNT-based sensors in the axial (strain) and transverse directions.

Sensor Type	ε_b_ (%)	∆R/R_0_ (%)		GF		GFν *
		Axial	Transverse	Axial	Transverse	Transverse
BP_DMF_/PI	2.7 ± 1.3	[17.5; 39.2]	[−20.8; −13.6]	[7.7; 9.4]	[−24.3; −16]	[−8.3; −5.5]
BP_ETOH_/PI	2.5 ± 0.6	[14.2; 23.7]	[−18.7; −12.4]	[7.2; 8.2]	[−17.9; −15.5]	[−6.1; −5.3]
Knocked down VA-CNT/PI	8.4	137	−27.8	16.4	−9.8	−3.3

* correction of GF values with the Poisson ratio, ν.

**Table 2:** Gauge factor (GF) values for CNT/polymer-based strain sensors. Acronyms: PMMA (polymethyl methacrylate), PSF (Polysulfone), PEO (polyethylene oxide).

Sensor Type	Fabrication Method	GF		References
		Axial	Transverse	
BP_DMF_/PI	Dispersion; Vacuum filtration	[7.7; 9.4]	[−24.3; −16]	Present work
BP_ETOH_/PI	Dispersion; Vacuum filtration	[7.2; 8.2]	[−17.9; −15.5]	Present work
Knocked down VA-CNT/PI	CVD; knock down	16.4	−9.8	[3]
MWCNT BP/epoxy	Dispersion; Vacuum filtration; incorporation within the matrix	≈0.85		[6]
MWCNT/epoxy	Solution mixing; isothermal curing	≈0.6		[27]
MWCNT/PMMA	Bulk mixing; melt processing	15.32 (1 wt.% MWCNT)		[28]
MWCNT/PSF	Solution mixing; mould casting (AC alignment)	2.68 (0.5 wt.% MWCNT)		[29]
MWCNT/PEO	Solution mixing; mould casting	50 (2.9 wt.% MWCNT)		[30]
